# Development and External Validation of Web-Based Models to Predict the Prognosis of Remnant Gastric Cancer after Surgery: A Multicenter Study

**DOI:** 10.1155/2019/6012826

**Published:** 2019-04-10

**Authors:** Qi-Yue Chen, Qing Zhong, Jun-Feng Zhou, Xian-Tu Qiu, Xue-Yi Dang, Li-Sheng Cai, Guo-Qiang Su, Dong-Bo Xu, Zhi-Yu Liu, Ping Li, Kai-Qing Guo, Jian-Wei Xie, Qiu-Xian Chen, Jia-Bin Wang, Teng-Wen Li, Jian-Xian Lin, Shuang-Ming Lin, Jun Lu, Long-Long Cao, Mi Lin, Ru-Hong Tu, Ze-Ning Huang, Ju-Li Lin, Wei Lin, Qing-Liang He, Chao-Hui Zheng, Chang-Ming Huang

**Affiliations:** ^1^Department of Gastric Surgery, Fujian Medical University Union Hospital, Fuzhou, China; ^2^Key Laboratory of Ministry of Education of Gastrointestinal Cancer, Fujian Medical University, Fuzhou, China; ^3^Department of Gastrointestinal Surgery, The First Affiliated Hospital of Fujian Medical University, Fuzhou, China; ^4^Department of Gastrointestinal Surgery and Gastrointestinal Surgery Research Institute, The Affiliated Hospital of Putian University, Putian, China; ^5^Department of General Surgery, Shanxi Provincial Cancer Hospital, Shanxi, China; ^6^Department of General Surgery Unit 4, Zhangzhou Affiliated Hospital of Fujian Medical University, Zhangzhou, China; ^7^Department of Gastrointestinal Surgery, The First Affiliated Hospital of Xiamen University, Xiamen, China; ^8^Department of Gastrointestinal Surgery, Longyan First Hospital Affiliated to Fujian Medical University, Longyan, China

## Abstract

**Background:**

Remnant gastric cancer (RGC) is a rare malignant tumor with poor prognosis. There is no universally accepted prognostic model for RGC.

**Methods:**

We analyzed data for 253 RGC patients who underwent radical gastrectomy from 6 centers. The prognosis prediction performances of the AJCC7th and AJCC8th TNM staging systems and the TRM staging system for RGC patients were evaluated. Web-based prediction models based on independent prognostic factors were developed to predict the survival of the RGC patients. External validation was performed using a cohort of 49 Chinese patients.

**Results:**

The predictive abilities of the AJCC8th and TRM staging systems were no better than those of the AJCC7th staging system (c-index: AJCC7th vs. AJCC8th vs. TRM, 0.743 vs. 0.732 vs. 0.744; P>0.05). Within each staging system, the survival of the two adjacent stages was not well discriminated (P>0.05). Multivariate analysis showed that age, tumor size, T stage, and N stage were independent prognostic factors. Based on the above variables, we developed 3 web-based prediction models, which were superior to the AJCC7th staging system in their discriminatory ability (c-index), predictive homogeneity (likelihood ratio chi-square), predictive accuracy (AIC, BIC), and model stability (time-dependent ROC curves). External validation showed predictable accuracies of 0.780, 0.822, and 0.700, respectively, in predicting overall survival, disease-specific survival, and disease-free survival.

**Conclusions:**

The AJCC TNM staging system and the TRM staging system did not enable good distinction among the RGC patients. We have developed and validated visual web-based prediction models that are superior to these staging systems.

## 1. Introduction

Remnant gastric cancer (RGC) encompasses all carcinomas arising in the remnant stomach following gastrectomy, irrespective of the histology of the primary disease (benign or malignant) or the extent of resection, the method of reconstruction, and nonrestriction of the time interval [1]. It has been reported that the incidence of RGC accounts for 1-3% of GC. Although the surgical treatment of patients with benign gastric diseases has decreased, the early detection of primary gastric cancer (PGC) and the improvement of the prognosis of patients with PGC have led to an increase of the incidence of RGC [2–4]. Compared with PGC, the diagnosis of RGC generally occurs at later stages, resulting in poor prognosis [3]. Therefore, the development of effective prognostic risk stratification for such rare patients has become a research hotspot.

The continuous update and improvement of the AJCC (American Joint Committee on Cancer) TNM staging are particularly conspicuous [5, 6], as this is generally accepted as an ideal prognostic evaluation system for PGC. However, the number of lymph nodes retrieved (RLNs) in RGC is lower than that in PGC, especially in patients with initial GC. Several studies have shown that the AJCC TNM staging of PGC cannot be fully applied to evaluate the prognosis of RGC, and the survival between stages is not completely distinguishable [7–9]. Recently, the rate of LN metastasis (LNR; the ratio of positive LNs to RLNs) is considered more suitable for evaluating LN status in PGC patients with fewer retrieved LNs than positive LNs. The TRM (tumor-ratio-metastasis) staging derived from the LNR is considered a good supplement to the TNM staging system [10–12]. However, the TRM staging system is still affected by the number of lymph nodes, and the value of RGC remains questionable.

Nomograms are multivariable survival prediction models based on individual patient characteristics and are widely used in the cancer field because they can provide individualized patient survival predictions [13, 14]. Although many nomograms of PGC have been reported [15–17], an RGC nomogram has not yet been reported. What is more, the calculation process of conventional nomogram is rather complex in clinical practice, which is not conducive to large-scale popularization and promotion, especially for patients. As we enter the era of personalized medicine, web-based nomograms are a way of visualizing nomograms that is more practical and easy to popularize [18, 19]. Therefore, based on multicenter data, the purpose of this study was to evaluate the prognosis prediction performance of the AJCC 7th TNM staging system, the AJCC 8th TNM staging system, and the TRM staging system and to establish a new web-based prognosis prediction model for RGC, thus providing an individualized, simple, and practical clinical prognostic assessment tool for this special group of GC patients.

## 2. Materials and Methods

### 2.1. Population and Covariates

For the purposes of the present study, RGC was defined as all newly diagnosed gastric cancers in the remnant stomach after partial gastrectomy regardless of the original disease. From January 2003 to January 2017, 322 patients with a history of gastrectomy were identified, including 283 patients from Fujian Medical University Union Hospital, the First Affiliated Hospital of Fujian Medical University, Zhangzhou Affiliated Hospital of Fujian Medical University, and Longyan First Affiliated Hospital of Fujian Medical University. 8 patients had been treated at the First Affiliated Hospital of Xiamen University, and 31 patients had been treated at Shanxi Provincial Cancer Hospital. The inclusion criteria were defined as follows: the presence of remnant GC, no combined malignancy, no preoperative chemotherapy and/or radiotherapy, no distant metastasis, and complete basic patient information. Exclusion criteria were defined as follows: histology showing a tumor type other than adenocarcinoma or a lack of either patient death or patient survival data. The remaining 253 patients who underwent surgery for RGC were included in the present study. The institutional review boards of all participating institutions approved the study.

The Roux-en-Y method was performed for reconstruction. Adjuvant chemotherapy was recommended to patients with Stage II or greater, according to the TNM stage for primary gastric cancer as described at that time. All patients received standard postoperative follow-up care from the participating centers, including visits every 3-6 months for the first 2 years, every 6-12 months from the 3rd to 5th year, and annually thereafter. All patients were observed until death or the final follow-up in May 2018.

#### 2.1.1. TNM and TRM Staging

Pathological (p) T and N stages were classified according to the 7th and 8th editions of the AJCC TNM staging system (T1: mucosa or submucosa, T2: proper muscle, T3: subserosa, T4a: serosa invasion, T4b: adjacent organ invasion; N0: no LN metastasis, N1: 1–2 metastatic LNs, N2: 3–6 metastatic LNs, N3a: 7–15 metastatic LNs, N3b: 16 or more metastatic LNs) [5, 6]. LNR was defined as the metastatic LN count divided by the RLN. The cutoff points for LNR were considered using the best cutoff approach and balancing the number of each classification as well as considering each patient's survival (using Kaplan–Meier curves). In the current study, novel cutoff values of LNR were determined as 0.3 and 0.6 based on overall survival using the software X-tile (Supplemental Figure 1). Node ratio (Nr) groups were categorized according to the cutoffs as follows: Nr0: 0; Nr1: 0 < LNR ≤ 0.3; Nr2: 0.3 < LNR ≤ 0.6; Nr3: 0.6 < LNR. A new staging system, named TRM, was constructed based on the same pT and M definitions from the 7th AJCC staging system (Supplemental Figure 2).

### 2.2. Statistical Analysis

Overall survival (OS) was defined as time from surgery to death from any cause, disease-specific survival (DSS) was defined as time from surgery to death from cancer, and disease-free survival (DFS) was defined as the time from surgery to the time of recurrence or death from any cause. Survival curves were estimated using the Kaplan–Meier method, and the log-rank test was used to determine significance.

Variables associated with OS, DSS, and DFS were selected using multivariate Cox regression models. Stepwise backward variable removal was applied to the multivariate model to identify the most accurate and parsimonious set of predictors [20]. For purposes of illustration and clinical applicability, three web-based nomograms were created based on the final regression model.

The performance of a prognostic system has been shown to be related to homogeneity (small differences in survival among patients in the same class within each system) and discriminatory ability (greater differences in survival among patients in different stages within each system). Harrell's c-index was used to measure the discriminatory ability of different prognostic systems [21, 22]. The likelihood ratio chi-square score was calculated using Cox regression to measure homogeneity; a higher likelihood ratio chi-square score indicates better homogeneity [23]. We used the Akaike information criterion (AIC) within the Cox regression model to compare performances between 2 prognostic systems; smaller AIC values represent better optimistic prognostic stratification [24]. We calculated the relative likelihood of two models using the formula: exp((AIC (model A) -AIC (model B))/2). The relative likelihood represents the probability that model A minimizes information as effectively as model B and can thus be interpreted as a P value for the comparison of both AIC values [25]. Calibration plots were generated to evaluate the performance characteristics of the prediction models. Receiver operating characteristic (ROC) curves were used to assess the discrimination power of the models. Decision curve analysis (DCA) was used to evaluate the clinical usefulness of the prediction models [26]. We also performed time-dependent receiver operating characteristics (time-dependent ROC) analysis to assess the discriminatory power of the prognosis model for time-dependent disease outcomes [27]. The Bayesian Information Criterion (BIC) was used to assess the overall prognostic performance of different prognostic systems via bootstrap-resampling analysis [28, 29]. External validation was performed using the Affiliated Hospital of Putian University validation cohort (PTAH; n=49; 2013-2017), which satisfied the aforementioned inclusion criteria.

All data were processed using SPSS 19.0 (SPSS Inc. Chicago, IL, USA) and R software (version 3.5.0). The R package “DynNom” was used to develop the web-based nomogram. All tests were two-sided with a significance level set to P<0.05.

## 3. Results

### 3.1. Demographic and Clinicopathologic Characteristics

The demographic and clinicopathologic characteristics of 253 RGC patients are summarized in Table 1. Among them, 79.1% of the patients were diagnosed with GC in the remnant stomach over 5 years after the primary gastrectomy. The mean age of the patients was 63.8 years (range 37-87 years), and the male-to-female ratio was 7.4:1. In total, 144 (56.9%) cases were located at the anastomosis site, and 109 (43.1%) cases were located at the nonanastomotic site. The average number of LNs examined was 16.1 (range 1–59), and 54.2% of patients had ≤15 RLN. Nearly half of patients (51%) received adjuvant chemotherapy after the RGC surgery. After a median follow-up of 64 months (1-235 months), the 5-year OS, the 5-year DSS, and the 5-year DFS after surgery were 48.39%, 53.73%, and 47.64%, respectively. A total of 87 patients in 253 patients had recurrence (34.4%), of which 24 patients had local recurrence (9.5%). 39 patients had peritoneal metastases (15.4%), and 59 patients had distant metastases (23.3%).

### 3.2. Survival: AJCC TNM and TRM Categories

**Figure 1:**
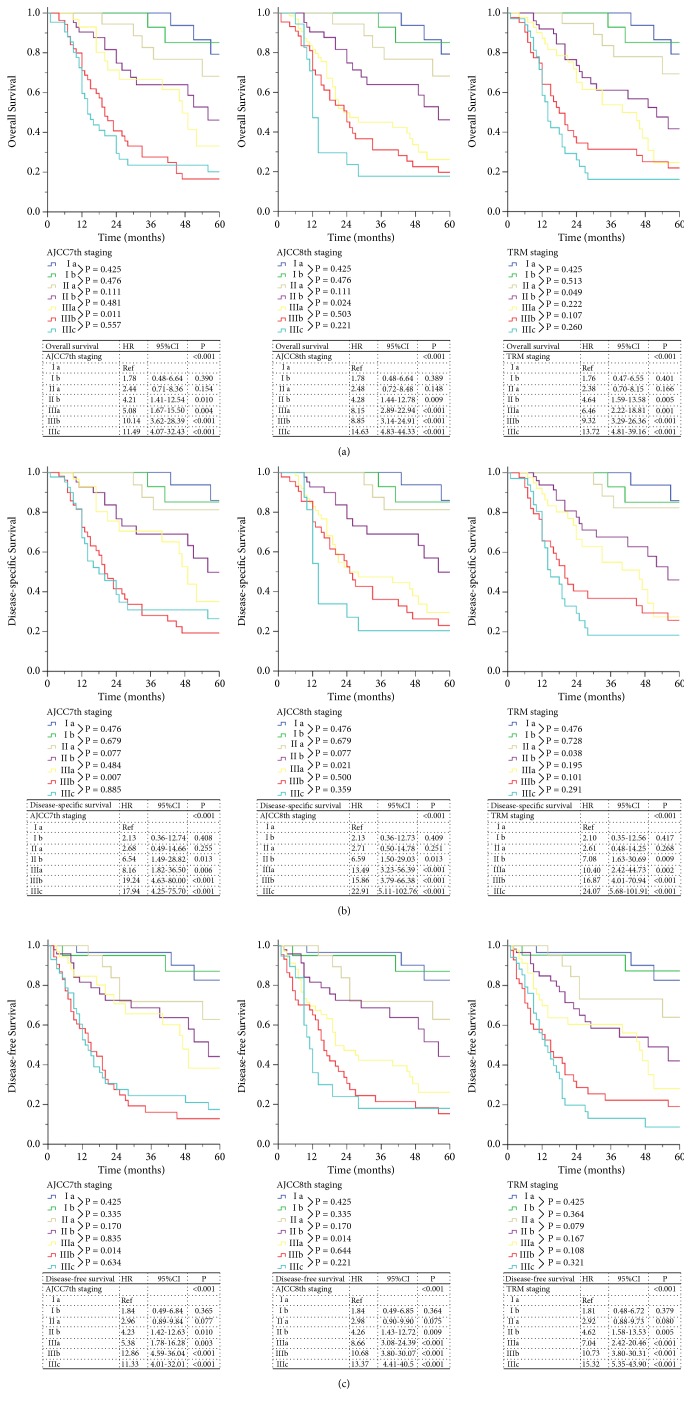
AJCC TNM staging (7th, 8th) versus TRM staging for remnant gastric cancer. (a) Overall survival, (b) disease-specific survival, (c) disease-free survival.

When comparing the 7th and 8th editions of the AJCC TNM staging system (Supplemental Table 1), 79.4% (n=201) of patients had the same staging in both staging systems, and the stages of 20.6% (n=52) of the patients differed between the systems. Furthermore, the comparison of the AJCC 7th staging system and the TRM staging system revealed that the tumor staging differed for 56 patients (22.1%) (Supplemental Table 2). The OS, DSS, and DFS of the three staging systems are shown in Figure 1. For the AJCC 7th staging system, the 5-year OS of Ia, Ib, IIa, IIb, IIIa, IIIb, and IIIc were 86.4%, 86.7%, 71.4%, 56.5%, 49.0%, 24.2%, and 24.1%, respectively (P<0.05). For the AJCC 8th staging system, the 5-year OS of Ia, Ib, IIa, IIb, IIIa, IIIb, and IIIc were 86.4%, 86.7%, 71.4%, 56.5%, 37.0%, 24.4%, and 20.0%, respectively (P<0.05). Moreover, the 5-year OS of Ia, Ib, IIa, IIb, IIIa, IIIb, and IIIc for the TRM staging system were 86.4%, 87.1%, 70.6%, 54.2%, 40.0%, 27.3%, and 18.8%, respectively (P<0.05). Further subgroup analysis showed no significant difference in OS between adjacent stages (P>0.05), except for IIIa and IIIb in the AJCC 7th staging system (P=0.011). Additionally, in the AJCC 8th staging system, other than IIb and IIIa (P=0.024), the OS of the adjacent groups could not be distinguished (P>0.05). In addition, there was no significant difference in the OS of adjacent stages (P>0.05), except for IIa and IIb in the TRM staging system (P=0.049). Similarly, there was no good distinction between two adjacent stages of the AJCC 8th and TRM staging systems for DSS and DFS.

### 3.3. Development of Prediction Models

**Figure 2:**
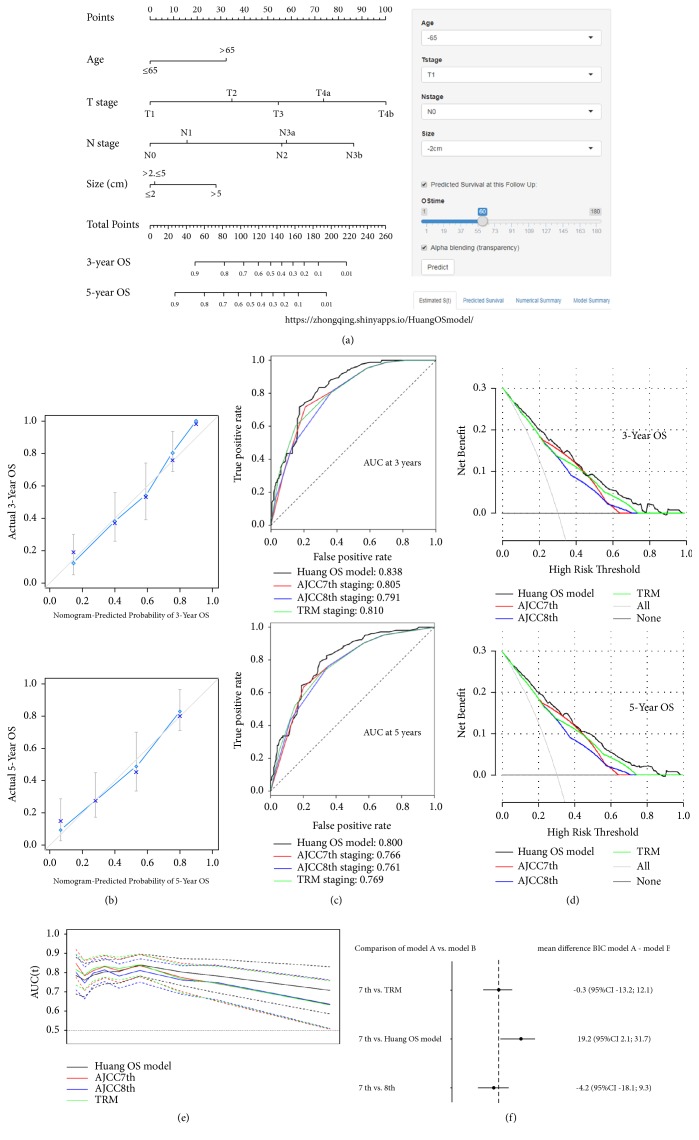
(a) The Huang OS model (a web-based OS nomogram) for predicting 3- and 5-year OS rates for RGC. The nomogram is used by summing the points identified on the point scale for each variable. The total points projected on the bottom scales indicate the probability of 3- and 5-year OS. The nomogram is available at https://zhongqing.shinyapps.io/HuangOSmodel/. To use this nomogram, choose the value for each variable and the predicted survival time; then press the “predict” button. (b) A calibration plot of the web-based nomogram for 3 years and 5 years. (c) The receiver operating characteristic (ROC) curves for the 3- and 5-year overall survival probability for the web-based nomogram and the 3 studied staging systems. (d) Decision curve analysis (DCA) for the 3-year OS and 5-year OS after surgery. The y-axis measures the net benefit. (e) Time-dependent ROC curves for the web-based nomogram and the 3 studied staging systems. The x-axis represents the year after surgery, and the y-axis represents the estimated area under the ROC curve for survival at the time of interest. (f) The results from a bootstrap analysis (1,000 samples): mean differences in Bayesian information criteria (BIC) with 95% confidence limits, including the web-based nomogram and the 3 studied staging systems.

**Figure 3:**
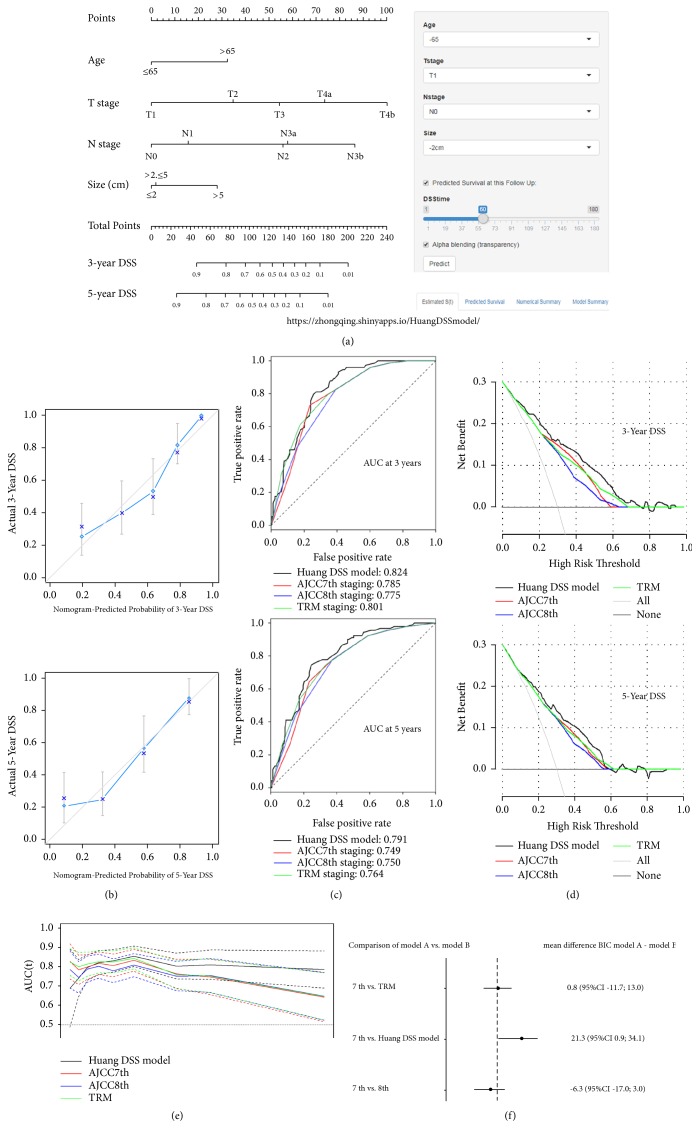
(a) The Huang DSS model (a web-based DSS nomogram) for predicting 3- and 5-year DSS rates for RGC. The nomogram is used by summing the points identified on the point scale for each variable. The total points projected on the bottom scales indicate the probability of 3- and 5-year DSS. The nomogram is available at https://zhongqing.shinyapps.io/HuangDSSmodel/. To use this nomogram, choose the value for each variable and the predicted survival time; then press the “predict” button. (b) A calibration plot of the web-based nomogram for 3 years and 5 years. (c) The receiver operating characteristic (ROC) curve for the 3- and 5-year disease-specific survival probability for the web-based nomogram and the 3 studied staging systems. (d) DCA for 3-year DSS and 5-year DSS after surgery. The y-axis measures the net benefit. (e) Time-dependent ROC curves for the web-based nomogram and the 3 studied staging systems. The x-axis represents the year after surgery, and the y-axis represents the estimated area under the ROC curve for survival at the time of interest. (f) The results from a bootstrap analysis (1,000 samples): mean differences in Bayesian information criteria (BIC) with 95% confidence limits, including the web-based nomogram and the 3 studied staging systems.

Univariate analysis demonstrated that several factors were related to OS (Table 2), including age (P=0.015), lymphovascular invasion (P<0.001), combined resection (P=0.029), histology (P=0.002), macroscopic type (P<0.001), tumor size (P=0.003), pT stage (P<0.001), pN stage (P<0.001), and adjuvant chemotherapy (P=0.008). After stepwise backward variable selection, age, tumor size, T stage, and N stage were found to be independent prognostic factors for OS in the multivariate analysis. Based on the above four variables, we developed a web-based nomogram (the Huang OS model, https://zhongqing.shinyapps.io/HuangOSmodel/) for the individualized prediction of the OS of RGC patients (Figure 2). Meanwhile, multivariate analysis also showed that the independent prognostic factors of DSS and DFS were age, tumor size, T stage, and N stage. Based on the final regression models, we also established two web-based nomograms (the Huang DSS model, https://zhongqing.shinyapps.io/HuangDSSmodel/, Figure 3, and the Huang DFS model, https://zhongqing.shinyapps.io/HuangDFSmodel/, Supplemental Figure 3) to accurately predict RGC patients' DSS and DFS.

### 3.4. Comparison of the Four Prognostic Classification Systems

The predictive ability of each prognostic system is compared in Table 3. Regardless of whether OS, DSS, or DFS was compared, there was no significant difference in the prognostic discriminability (c-index) between the AJCC 8th staging and TRM staging systems when compared with the AJCC 7th staging system (P>0.05), while the Huang model was superior to the 7th AJCC staging system when comparing the c-index (Huang OS model 0.774 vs. AJCC 7th staging 0.743, p = 0.037; Huang DSS model 0.773 vs. AJCC 7th staging 0.742, p = 0.032; Huang DFS model 0.744 vs. AJCC 7th staging 0.710, p = 0.021). AIC analysis showed that the AJCC 7th staging system, the AJCC 8th staging system, and the TRM staging system possessed similar goodness of fits for OS, DSS, and DFS (relative likelihood >0.05), while the Huang model had a better goodness of fit than did the AJCC 7th staging system (relative likelihood <0.05). The Huang model also had better performance based on the likelihood ratio chi-square. Moreover, the stratification analysis confirmed that regardless of whether the number of LNs examined was more than 15 or not, the prediction performances of the web-based nomograms were better than those of the other three staging systems (Supplemental Table 3).

### 3.5. Validation

The nomogram calibration plot demonstrated good agreement between the predicted and observed survival rates for each of OS, DSS, and DFS (Figures 2(b) and 3(b) and Supplemental Figure 3(B)). The ROC curves showed that the prediction accuracy of the Huang model is better than those of the AJCC staging and TRM staging systems for OS, DSS, and DFS (Figures 2(c) and 3(c) and Supplemental Figure 3(C)). In addition, the DCA was used to evaluate and compare the clinical usefulness of various prognostic models (Figures 2(d) and 3(d) and Supplemental Figure 3(D)), and the results showed that the Huang model provided a better net benefit than the other three staging systems at the same probability threshold. Using time-dependent ROC curves, we compared the continuous trends of the survival hazard ratio for each staging system. As shown in Figures 2(e) and 3(e) and Supplemental Figure 3(E), the Huang model proved superior to the other three staging systems over time. BIC, a criterion that accurately considers the number of parameters included in the models, was used to assess the overall prognostic performance of the various prognostic systems. As shown in Figures 2(f) and 3(f) and Supplemental Figure 3(F), there was no significant difference between the AJCC 7th staging system and TRM staging system in the bootstrap analysis; neither was there a difference between the AJCC 7th and the AJCC 8th staging systems. However, when compared to the AJCC 7th staging system, the multivariate “Huang model” appeared to have a slight but obvious advantage. Supplemental Figure 4 illustrates the discrepancies between the two prediction methods. For each AJCC 7th stage grouping, histograms of nomogram-predicted probabilities are presented, showing the advantage of the web-based nomogram over the AJCC 7th stage groupings.

In the external validation cohort, the calibration curve of the web-based nomogram (Supplemental Figure 5) also showed good agreement between the predicted and observed outcomes. The predictive ability of the web-based prognostic model was also better than that of other 3 different staging systems in the validation cohort (Supplemental Table 5).

## 4. Discussion

Due to the rarity of RGC, the recent guidelines for gastric cancer, including the Japanese gastric cancer guideline, the NCCN guideline, and the ESMO guideline, mention only in their definition of RGC the tumor location in the remnant stomach and the regional LN [1, 30–32]. There is no consensus on the best prognostic staging system for RGC. The staging system of PGC is still used for RGC in clinical practice at the present time.

However, previous studies have reported that RGCs have different prognostic characteristics than PGCs [2, 4, 33, 34], and the survival of RGC patients showed significant heterogeneity. Therefore, knowing how to develop an accurate risk stratification is very important for RGC patients and clinicians. The AJCC TNM staging system, one of the most important prognostic staging systems, has become an important reference for clinical treatment decision-making and assessing the prognosis of PGC. With the cooperation of and promotion by the AJCC, UICC, and IGCA, the most recent edition of the AJCC TNM staging system, the AJCC 8th edition, was published in 2017 through the accumulation and analysis of big data. However, the practicability of the AJCC 8th staging system in RGC has not been reported. The results of this study suggest that the AJCC 8th staging system is not a significant improvement over the AJCC 7th staging system (c-index: AJCC 7th staging vs. AJCC 8th staging, OS: 0.743 vs. 0.732; DSS: 0.742 vs. 0.731; DFS: 0.710 vs. 0.700). Several studies have demonstrated that examining at least 16 LNs can provide better predictions of the prognosis of GC patients [35, 36]. Nevertheless, the local anatomy and lymphatic flow of RGC are changed by the primary operation, which results in a lower number of LN being examined and stage migration. It has been recognized that the TRM staging system can help doctors to distinguish the prognosis of PGC patients regardless of the number of LNs examined [12, 37]. However, we found that, compared with the 7th AJCC staging system, the TRM staging system did not significantly improve the ability to distinguish the prognosis of patients with RGC (c-index: AJCC 7th staging vs. TRM staging, OS: 0.743 vs. 0.744; DSS: 0.742 vs. 0.754; DFS: 0.710 vs. 0.714). More importantly, regardless of whether OS, DSS, or DFS was examined, there was no significant difference between most pairs of adjacent stages in each staging system (P>0.05).

It is possible that the staging system of PGC may not be sufficiently accurate to evaluate the prognosis of patients with RGC. Therefore, three web-based prediction models for predicting the OS, DSS, and DFS of RGC patients were established in combination with the prognostic factors. When compared with three other staging systems, the web-based prediction model was shown to improve the prediction performance in terms of the discriminatory ability (c-index), predictive homogeneity (likelihood ratio chi-square), predictive accuracy (AIC, BIC), and model stability (time-dependent ROC curves). The calibration curve and ROC curve also show that the web-based prediction models provide good prediction accuracy. In addition, the DCA also demonstrated that the Huang model could provide a greater net benefit and clinical value than the other three staging systems. Moreover, the stratified analysis showed that regardless of whether the number of LNs examined was more than 15 or not, the web-based model remained superior to the other three staging systems. External validation in the PTAH validation cohort demonstrated good discrimination power (Harrell's c-index, 0.780, 0.822 and 0.700). Calibration using the PTAH validation cohort demonstrated that the actual survival corresponds closely to the predicted survival.

Compared to traditional staging systems, risk grouping, or nomograms, the web-based prediction model can be accessed using a personal computer or website-enabled cellular phone, which is convenient for clinicians, such that they can use it in real time to better inform the patient's prognosis, providing great convenience and practicability. With the development of medical information technology, the web-based prediction model is also easier to popularize. Based on multicenter data, we developed the first web-based prediction model for predicting survival in RGC. This model can guide doctors in developing a personalized follow-up strategy and can become one part of a standardized disease management after RGC surgery.

At present, in East Asian countries including China, standard D2 lymph node dissection plus postoperative adjuvant chemotherapy has been the standard treatment for advanced GC for many years [32]. Based on two important phase III clinical studies, CLASSIC [38] and ACTS-GC [39], it has been confirmed that postoperative chemotherapy can improve the survival of advanced PGC. However, RCTs for gastric cancer patients often use RGC as an exclusion criterion, and there are no prospective studies for the chemotherapy of RGC. Although adjuvant chemotherapy is not an independent prognostic factor for RGC patients in this study, based on existing evidence of RGC [4], we still believe that adjuvant chemotherapy is an important therapeutic component for patients with advanced RGC. We look forward to further confirming the impact of chemotherapy on RGC survival through a multicenter prospective study.

Because GC resection is often accompanied by extensive lymphadenectomy, the route of LN metastasis in RGC patients with initial malignant disease surgery may be different from that of patients after initial benign disease surgery. Therefore, whether there is a difference in the prognosis between initial surgery for benign disease and malignant disease remains controversial. Previous studies have shown a contradictory result between the malignant and benign groups (Supplemental Table 6) [40–44]. We combined the data, which revealed that there was no significant difference between the two groups of patients in 5-year OS (45.2% vs. 41.7%). The results of this study also showed that the 5-year OS of RGC patients with initial malignant disease surgery was 48.61%, and the 5-year OS of RGC patients with initial benign disease surgery was 48.26%; there was no significant difference between the two groups (p=0.709). Although RGC in patients with primary malignancy appears earlier than in patients with primary benign disease [45], the univariate and multivariate Cox analysis in our study showed that the type of the primary disease was not an independent prognostic factor. Therefore, we believe that the web-based prediction model we have established is still applicable to most RGC patients. More in-depth analysis of prognosis of RGC between first operation for benign disease and that for malignant disease by enlarging data size is needed.

RGC is rare; this study not only assessed the prognosis predictive ability of the AJCC 7th, AJCC 8th, and TRM staging systems in patients with RGC but also established an interactive web-based prediction model for predicting the survival of RGC patients. However, our study is not without limitations. First, this study is a multicenter retrospective study, and potential bias is inevitable. Previous literature reported that many immunoinflammatory markers (e.g., NLR, PLR, and LMR) have prognostic value in PGC [46–48]. However, due to the rarity of RGC, there is no report on the influence of immunoinflammatory markers on the prognosis of RGC. Because of the limitations of our retrospective study, we did not conduct in-depth analysis of preoperative biochemical parameters. In addition, this study did not include the increasingly recognized prognostic tissue-based biomarkers (e.g., MSI) in the analysis [49, 50]. What is more, although the results obtained by incorporating data from several Chinese centers were highly universal and applicable, the number of cases was still relatively small. In addition, because of the differences in treatment patterns between the East and the West, Chinese gastric cancer patients rarely receive preoperative therapy compared with those in Europe and the United States. As is already known, preoperative chemotherapy has a chemotherapy-related downstages effect. Postoperative pathological T stage (ypT) and pathological N (ypN) stage of gastric cancer patients after preoperative therapy may have different effects on prognosis compared with the same pathological T stage (pT) and pathological N stage (pN) of patients without preoperative chemotherapy. Therefore, we did not include patients with neoadjuvant therapy to avoid the impact of neoadjuvant therapy on the accuracy of the results. Furthermore, we adopted any statistical methods to validate the web-based model and prove its excellent predictive performance; these user-friendly web-based models showed precise discriminative ability both in the development cohort and in the external validation cohort, supporting the generalizability of these nomograms. However, the sample size for validation study was small and patients were enrolled from single institute. More patient from multiple institutes might be needed for validation study. What is more, we continue to need validation from Western big data because of differences in socioeconomic status and diagnosis levels between nations. We look forward to the validation and even to the improvement of the web-based prediction model by further selecting larger and more representative data for RGC cases from a greater number of countries worldwide.

In summary, the various AJCC TNM and TRM staging systems did not distinguish the survival of patients with RGC well. We have developed and validated user-friendly web-based prediction models that are superior to the currently used staging systems. These models can accurately predict the prognosis of RGC patients and guide clinical practice.

## Figures and Tables

**Table 1:** Clinicopathologic description of all remnant gastric cancer patients.

Variable	No. of Patients	%
*Age (years) (Mean ± SD)*	63.8 ± 9.6	
>65	147	58.1
≤65	106	41.9
*Sex*		
Male	223	88.1
Female	30	11.9
*Family history*		
No	238	94.1
Yes	15	5.9
*Interval (year)*		
≤5	53	20.9
5-10	28	11.1
>10	172	68.0
*Previous disease*		
Benign	165	65.2
Malignant	88	34.8
*Previous operation type *		
Distal gastrectomy	244	96.4
Proximal gastrectomy	9	3.6
*Reconstruction*		
Billroth I	71	28.1
Billroth II	168	66.4
Roux-en-Y	5	2.0
Esophageal gastric remnant anastomosis	9	3.6
*Comorbidity*		
No	155	61.3
Yes	98	38.7
*ASA score*		
I-II	215	85.0
III-IV	38	15.0
*Tumor location*		
Anastomosis	144	56.9
Nonanastomotic site	109	43.1
*Approach*		
Open	174	68.8
Laparoscopic	79	31.2
*Combined resection*		
No	207	81.8
Yes	46	18.2
*Lymphvascular invasion*		
No	159	62.8
Yes	94	37.2
*Histology*		
Differentiated	110	43.5
Undifferentiated	143	56.5
*Macroscopic type*		
EGC	34	13.4
AGC, Borrmann 1-3	193	76.3
AGC, Borrmann 4	26	10.3
*Size (cm) (Mean ± SD)*	4.5 ± 2.1	
≤2	44	17.4
2-5	128	50.6
>5	81	32.0
*pT-stage *		
T1	34	13.4
T2	30	11.9
T3	55	21.7
T4a	91	36.0
T4b	43	17.0
*pN-stage*		
N0	110	43.5
N1	45	17.8
N2	49	19.4
N3a	41	16.2
N3b	8	3.2
*Positive LN count (Mean ± SD)*	3.4 ± 5.3	
*Retrieved LN count (Mean ± SD)*	16.1 ± 9.6	
≤ 15	137	54.2
>15	116	45.8
*LNR*		
0	110	43.5
>0,0.3	69	27.3
>0.3,0.6	42	16.6
>0.6	32	12.6
*AJCC7th staging*		
Ia	31	12.3
Ib	20	7.9
IIa	23	9.1
IIb	48	19.0
IIIa	35	13.8
IIIb	53	20.9
IIIc	43	17.0
*AJCC8th staging*		
Ia	31	12.3
Ib	20	7.9
IIa	23	9.1
IIb	48	19.0
IIIa	68	26.9
IIIb	44	17.4
IIIc	19	7.5
*Complication*		
No	148	58.5
Yes	105	41.5
*Adjuvant Chemotherapy*		
No	108	42.7
Yes	145	57.3
*Radiotherapy*		
No	250	98.8
Yes	3	1.2

*Abbreviations*. SD: standard deviation; ASA: American Society of Anesthesiologists; LN: lymph node; EGC: early gastric cancer; AGC: advanced gastric cancer; Family history: family history of gastric cancer; Interval: interval between gastrectomy and remnant gastric cancer.

**Table 2:** Multivariable analyses for overall survival, disease-specific survival, and disease-free survival.

	Overall survival			Disease-specific survival			Disease-free survival		
	Multivariate Model†			Multivariate Model†			Multivariate Model†		

Variable	HR	95% CI	P	HR	95% CI	P	HR	95% CI	P

*Age (year)*			<0.001			0.008			0.005
>65	Ref			Ref			Ref		
≤65	2.05	1.36-3.08	<0.001	1.86	1.18-2.93	0.008	1.77	1.20-2.64	0.005
*Size (cm)*			0.012			0.005			0.006
≤2	Ref			Ref			Ref		
2-5	1.05	0.50-2.18	0.905	1.03	0.43-2.51	0.943	1.22	0.58-2.57	0.603
>5	1.87	1.20-2.93	0.006	2.17	0.95-4.93	0.065	1.96	1.28-3.01	0.002
*T-stage *			<0.001			<0.001			<0.001
T1	Ref			Ref			Ref		
T2	2.19	0.67-7.16	0.195	3.08	0.64-14.85	0.161	2.54	0.78-8.28	0.122
T3	3.40	1.11-10.49	0.033	4.36	0.95-19.93	0.058	4.44	1.45-13.62	0.009
T4a	5.21	1.76-15.47	<0.001	7.40	1.68-32.58	0.008	6.19	2.07-18.48	0.001
T4b	9.36	2.98-29.44	<0.001	16.00	3.46-74.02	<0.001	12.87	4.05-40.94	<0.001
*N-stage*			<0.001			<0.001			0.001
N0	Ref			Ref			Ref		
N1	1.42	0.78-2.60	0.258	1.39	0.71-2.71	0.337	1.32	0.74-2.34	0.349
N2	3.49	1.86-6.53	<0.001	3.49	1.80-6.80	<0.001	2.43	1.34-4.42	0.004
N3a	3.59	1.96-6.56	<0.001	3.70	1.88-7.27	<0.001	2.81	1.58-5.01	<0.001
N3b	6.72	2.31-19.57	<0.001	6.06	1.85-19.87	0.003	3.18	1.13-9.00	0.029

†Adjusted for age, sex, BMI, family history of gastric cancer, interval between gastrectomy and remnant gastric cancer, previous disease, previous operation type, reconstruction, comorbidity, complication, ASA score, tumor location, lymphovascular invasion, approach, combined resection, histology, macroscopic type, retrieved lymph node, complication, and adjuvant chemotherapy.

**Table 3:** Comparison of the prognostic performances of the 3 studied staging systems and the web-based prognostic model.

Overall survival	AJCC7th staging	AJCC8th staging	TRM staging	Huang OS model
Harrell's C index*∗*	0.743 (0.702-0.785)	0.732 (0.689-0.774)	0.744 (0.704-0.785)	0.774 (0.733-0.815)
P value*∗∗*		0.254	0.927	0.037
AIC†	1060.08	1064.41	1060.46	1042.71
Relative likelihood††		0.115	0.827	<0.001
Likelihood ratio chi-square‡	65.86	61.53	65.48	91.25

Disease-specific survival	AJCC7th staging	AJCC8th staging	TRM staging	Huang DSS model

Harrell's C index*∗*	0.742 (0.700-0.784)	0.731 (0.687-0.775)	0.754 (0.714-0.796)	0.773 (0.730-0.817)
P value*∗∗*		0.408	0.290	0.032
AIC†	872.37	878.3	871.63	864.15
Relative likelihood††		0.052	0.691	0.016
Likelihood ratio chi-square‡	70.32	64.39	71.06	85.54

Disease-free survival	AJCC7th staging	AJCC8th staging	TRM staging	Huang DFS model

Harrell's C index*∗*	0.710 (0.667-0.754)	0.700 (0.656-0.744)	0.714 (0.672-0.755)	0.744 (0.702-0.787)
P value*∗∗*		0.397	0.703	0.021
AIC†	1117.27	1123.18	1118.11	1109.79
Relative likelihood††		0.052	0.657	0.024
Likelihood ratio chi-square‡	72.49	64.58	71.66	84.98

AIC: Akaike information criterion.

*∗* A higher Harrell's C index indicates higher discriminative ability.

*∗∗* P value of Harrell's C index (compare with AJCC7th staging system).

† Smaller AIC values indicate better optimistic prognostic stratification.

†† The relative likelihood could be interpreted as a P value for the comparison of both AIC values (compare with AJCC7th staging system).

‡ A higher likelihood ratio chi-square score means better homogeneity.

## Data Availability

The datasets used and/or analyzed during the current study are available from the corresponding author on reasonable request.

## Supplementary Materials

Supplementary Materials Supplemental Table 1. Correlation between the 7th edition and 8th edition AJCC staging systems. Supplemental Table 2. Correlation between the 7th edition AJCC staging system and the TRM staging system. Supplemental Table 3. Comparison of the prognostic performances of the 3 studied staging systems and the web-based prognostic model based on (A) a retrieved LN count ≤15 and (B) a retrieved LN count ≤15. Supplemental Table 4. Clinicopathologic description of remnant gastric cancer patients in the validation cohort. Supplemental Table 5. Comparison of the prognostic performances of the 3 studied staging systems and the web-based prognostic model in the validation cohort. Supplemental Table 6. Five-year postoperative survival rates in remnant gastric cancer between initial surgery for benign disease and initial surgery for malignant disease in previous studies. Supplemental Figure 1. X-tile analysis identifying the best cutoff points for LNR; LNR, metastatic lymph node ratio. Supplemental Figure 2. (A) The AJCC 7th staging system, (B) the TRM staging system, (C) the AJCC 8th staging system. Supplemental Figure 3. (A) The Huang DFS model (a web-based nomogram) for predicting the 3- and 5-year DFS rates for RGC. The nomogram is used by summing the points identified on the point scale for each variable. The total points projected on the bottom scales indicate the probability of 3- and 5-year DFS. The nomogram is available at https://zhongqing.shinyapps.io/Huang DFS model/. To use this nomogram, choose the value for each variable and the predicted survival time; then press the “predict” button. (B) A calibration plot of the web-based nomogram for 3 years and 5 years. (C) The receiver operating characteristic (ROC) curve for the 3- and 5-year disease-specific probability of the web-based nomogram and the 3 studied staging systems. (D) DCA for 3-year DFS and 5-year DFS after surgery. The y-axis measures the net benefit. (E) Time-dependent ROC curves for the web-based nomogram and the 3 studied staging systems. The x-axis represents the year after surgery, and the y-axis represents the estimated area under the ROC curve for survival at the time of interest. (F) The results from a bootstrap analysis (1,000 samples): mean differences in Bayesian information criteria (BIC) with 95% confidence limits for the web-based nomogram and the 3 studied staging systems. Supplemental Figure 4. Distribution of the nomogram predictions within each AJCC 7th stage grouping. The nomogram's improved predictive ability is illustrated by the heterogeneity of the nomogram predictions. (A) Overall survival, (B) disease-specific survival, (C) disease-free survival. Supplemental Figure 5. Nomogram calibration plots using the external validation cohort. (A) Overall survival, (B) disease-specific survival, (C) disease-free survival.
