# Supplementary material for: Development and External Validation of Web-Based Models to Predict the Prognosis of Remnant Gastric Cancer after Surgery: A Multicenter Study
Source: J Oncol. 2019 Apr 10;2019:6012826. doi: 10.1155/2019/6012826 (PMC6481035; doi:10.1155/2019/6012826)
Supplement: Supplementary Materials — Supplemental Table 1. Correlation between the 7th edition and 8th edition AJCC staging systems. Supplemental Table 2. Correlation between the 7th edition AJCC staging system and the TRM staging system. Supplemental Table 3. Comparison of the prognostic performances of the 3 studied staging systems and the web-based prognostic model based on (A) a retrieved LN count ≤15 and (B) a retrieved LN count ≤15. Supplemental Table 4. Clinicopathologic description of remnant gastric cancer patients in the validation cohort. Supplemental Table 5. Comparison of the prognostic performances of the 3 studied staging systems and the web-based prognostic model in the validation cohort. Supplemental Table 6. Five-year postoperative survival rates in remnant gastric cancer between initial surgery for benign disease and initial surgery for malignant disease in previous studies. Supplemental Figure 1. X-tile analysis identifying the best cutoff points for LNR; LNR, metastatic lymph node ratio. Supplemental Figure 2. (A) The AJCC 7th staging system, (B) the TRM staging system, (C) the AJCC 8th staging system. Supplemental Figure 3. (A) The Huang DFS model (a web-based nomogram) for predicting the 3- and 5-year DFS rates for RGC. The nomogram is used by summing the points identified on the point scale for each variable. The total points projected on the bottom scales indicate the probability of 3- and 5-year DFS. The nomogram is available at https://zhongqing.shinyapps.io/Huang DFS model/. To use this nomogram, choose the value for each variable and the predicted survival time; then press the “predict” button. (B) A calibration plot of the web-based nomogram for 3 years and 5 years. (C) The receiver operating characteristic (ROC) curve for the 3- and 5-year disease-specific probability of the web-based nomogram and the 3 studied staging systems. (D) DCA for 3-year DFS and 5-year DFS after surgery. The y-axis measures the net benefit. (E) Time-dependent ROC curves for the web-based nomogram [file 6012826.f1.pdf]

Supplemental Table 1. Correlation between the 7th edition and 8th edition AJCC staging systems.

		AJCC 8th staging							Total
		Ia	Ib	IIa	IIb	IIIa	IIIb	IIIc	
AJCC 7th staging	Ia	31	0	0	0	0	0	0	31(12.3%)
	Ib	0	20	0	0	0	0	0	20(7.9%)
	IIa	0	0	23	0	0	0	0	23(9.1%)
	IIb	0	0	0	48	0	0	0	48(19.0%)
	IIIa	0	0	0	0	35	0	0	35(13.8%)
	IIIb	0	0	0	0	33	19	1	53(20.9%)
	IIIc	0	0	0	0	0	25	18	43(17.0%)
Total		31(12.3%)	20(7.9%)	23(9.1%)	48(19.0%)	68(26.9%)	44(17.4%)	19(7.5%)	253(100.0%)

Supplemental Table 2. Correlation between the 7th edition AJCC staging system and the TRM staging system

		TRM staging							Total
		Ia	Ib	IIa	IIb	IIIa	IIIb	IIIc	
AJCC 7th staging	Ia	31	0	0	0	0	0	0	31(12.3%)
	Ib	0	20	0	0	0	0	0	20(7.9%)
	IIa	0	1	21	1	0	0	0	23(9.1%)
	IIb	0	0	1	45	2	0	0	48(19.0%)
	IIIa	0	0	0	11	23	1	0	35(13.8%)
	IIIb	0	0	0	1	19	28	5	53(20.9%)
	IIIc	0	0	0	0	1	13	29	43(17.0%)
Total		31(12.3%)	21(8.3%)	22(8.7%)	58(22.9%)	45(17.8%)	42(16.6%)	34(13.4%)	253(100.0%)

Supplemental Table 3. Comparison of the prognostic performances of the 3 studied staging systems and the web-based prognostic model based on (A) a retrieved LN count ≤15; (B) a retrieved LN count ≤15.

		**(A) Retrieved LN count ≤15**		
**Overall Survival**	**AJCC7th staging**	**AJCC8th staging**	**TRM staging**	**Huang OS model**
**Harrell's C statistic***	0.741(0.684-0.800)	0.710(0.647-0.772)	0.742（0.687-0.797）	0.775(0.718-0.832)
**P value****		0.120	0.979	0.044
**AIC†**	519.71	524.45	516.82	507.85
**Relative likelihood††**		0.093	0.236	0.003
**Likelihood ratio chi-square‡**	31.87	27.13	34.76	51.73
**Disease-Specific Survival**	**AJCC7th staging**	**AJCC8th staging**	**TRM staging**	**Huang DSS model**
**Harrell's C statistic***	0.751(0.714-0.827)	0.731(0.670-0.793)	0.754（0.721-0.827）	0.790(0.731-0.850)
**P value****		0.148	0.812	0.039
**AIC†**	443.78	450.26	439.54	428.77
**Relative likelihood††**		0.039	0.120	0.001
**Likelihood ratio chi-square‡**	42.35	35.87	46.6	65.36
**Disease-Free Survival**	**AJCC7th staging**	**AJCC8th staging**	**TRM staging**	**Huang DFS model**
**Harrell's C statistic***	0.739(0.685-0.794)	0.717(0.660-0.774)	0.743（0.691-0.795）	0.754(0.698-0.810)
**P value****		0.295	0.839	0.602
**AIC†**	544.64	551.16	542.02	538.32
**Relative likelihood††**		0.038	0.270	0.042
**Likelihood ratio chi-square‡**	39.97	33.45	42.59	54.29
		**(B) Retrieved LN count >15**		
**Overall Survival**	**AJCC7th staging**	**AJCC8th staging**	**TRM staging**	**Huang OS model**
**Harrell's C statistic***	0.748(0.714-0.823)	0.759(0.724-0.835)	0.769（0.735-0.844）	0.785(0.721-0.848)
**P value****		0.650	0.319	0.041
**AIC†**	393.71	390.04	388.05	382.67
**Relative likelihood††**		0.160	16.945	0.004
**Likelihood ratio chi-square‡**	38.38	40.05	37.04	46.38
**Disease-Specific Survival**	**AJCC7th staging**	**AJCC8th staging**	**TRM staging**	**Huang DSS model**
**Harrell's C statistic***	0.754(0.697-0.811)	0.753(0.694-0.812)	0.756（0.697-0.815）	0.777(0.705-0.849)
**P value****		0.924	0.748	0.109
**AIC†**	308.1	307.98	307.7	302.54
**Relative likelihood††**		0.942	0.819	0.062
**Likelihood ratio chi-square‡**	28.06	28.68	29.46	32.02
**Disease-Free Survival**	**AJCC7th staging**	**AJCC8th staging**	**TRM staging**	**Huang DFS model**
**Harrell's C statistic***	0.734(0.671-0.797)	0.728(0.663-0.793)	0.735（0.668-0.802）	0.762(0.692-0.831)
**P value****		0.812	0.957	0.126
**AIC†**	416	417.17	417.91	408.71
**Relative likelihood††**		0.557	0.385	0.026
**Likelihood ratio chi-square‡**	34.95	33.79	33.04	41.22

AIC, Akaike information criterion.

* A higher Harrell's C statistics indicates higher discriminative ability; ** P value (compare with AJCC7th staging system); † Smaller AIC values indicate better optimistic prognostic stratification; ‡ A higher likelihood ratio chi-square score means better homogeneity; ‡ ‡ The relative likelihood could be interpreted as a p value for the comparison of both AIC (compare with AJCC7th staging system)

Supplemental Table 4. Clinicopathologic description of remnant gastric cancer patients in the validation cohort.

Variable	No. of Patients	%
**Age(years) (Mean ± SD)**	69.7± 8.2	
>65	17	34.7
≤65	32	65.3
**Sex**		
Male	42	85.7
Female	7	14.3
**Interval (year)**		
≤5	7	14.3
5-10	5	10.2
>10	37	75.5
**Previous disease**		
Benign	36	73.5
Malignant	13	26.5
**Size (cm) (Mean ± SD)**	5.0±2.9	
≤2	6	12.2
2-5	27	55.1
>5	16	32.7
**T-stage**		
T1	11	22.4
T2	2	4.1
T3	2	4.1
T4a	32	65.3
T4b	2	4.1
**N-stage**		
N0	23	46.9
N1	4	8.2
N2	12	24.5
N3	10	20.4
**Positive LN count (Mean ± SD)**	3.2±4.0	
**Retrieved LN count (Mean ± SD)**	14.0±9.0	
≤ 15	28	57.1
>15	21	42.9
**LNR**		
0	23	46.9
>0,0.3	10	20.4
>0.3,0.6	7	14.3
>0.6	9	18.4

**TRM staging**		
Ia	11	22.4
Ib	2	4.1
IIa	1	2.0
IIb	10	20.4
IIIa	9	18.4
IIIb	7	14.3
IIIc	9	18.4
**AJCC7th staging**		
Ia	11	22.4
Ib	2	4.1
IIa	1	2.0
IIb	9	18.4
IIIa	4	8.2
IIIb	10	20.4
IIIc	12	24.5
**AJCC8th staging**		
Ia	11	22.4
Ib	2	4.1
IIa	1	2.0
IIb	9	18.4
IIIa	11	22.4
IIIb	3	6.1
IIIc	12	24.5
**Adjuvant Chemotherapy**		
No	27	55.1
Yes	22	44.9

Abbreviations: SD, standard deviation; LN, Lymph node; ; Interval, Interval between gastrectomy and remnant gastric cancer.

Supplemental Table 5. Comparison of the prognostic performances of the 3 studied staging systems and the web-based prognostic model in the validation cohort.

**Overall Survival**	**AJCC7th staging**	**AJCC8th staging**	**TRM staging**	**Huang OS model**
**Harrell's C statistic***	0.721(0.594-0.847)	0.707(0.572-0.842)	0.723（0.584-0.863）	0.780(0.668-0.893)
**P value****		0.619	0.927	0.043
**AIC†**	127.95	131.05	131.2	121.75
**Relative likelihood††**		0.212	0.197	0.045
**Likelihood ratio chi-square‡**	16.54	13.44	13.29	25.73
**Disease-Specific Survival**	**AJCC7th staging**	**AJCC8th staging**	**TRM staging**	**Huang DSS model**
**Harrell's C statistic***	0.763(0.618-0.909)	0.745(0.599-0.891)	0.755（0.603-0.907）	0.822(0.701-0.942)
**P value****		0.398	0.790	0.047
**AIC†**	107.75	110.32	110.31	101.75
**Relative likelihood††**		0.277	0.278	0.050
**Likelihood ratio chi-square‡**	18.95	16.38	16.39	26.71
**Disease-Free Survival**	**AJCC7th staging**	**AJCC8th staging**	**TRM staging**	**Huang DFS model**
**Harrell's C statistic***	0.671(0.524-0.818)	0.649(0.499-0.798)	0.665（0.513-0.817）	0.700(0.551-0.848)
**P value****		0.251	0.834	0.486
**AIC†**	144.77	145.33	145.64	140.57
**Relative likelihood††**		0.756	0.647	0.122
**Likelihood ratio chi-square‡**	12.36	11.8	11.49	17.13

AIC, Akaike information criterion.

* A higher Harrell's C statistics indicates higher discriminative ability

** P value (compare with AJCC7th staging system)

† Smaller AIC values indicate better optimistic prognostic stratification.

‡ A higher likelihood ratio chi-square score means better homogeneity.

‡ ‡ The relative likelihood could be interpreted as a p value for the comparison of both AIC (compare with AJCC7th staging system)

Supplemental Table 6. Five-year postoperative survival rates in remnant gastric cancer between initial surgery for benign disease and initial surgery for malignant disease in previous studies

Author, year	Initially benign	Initially malignant	p value
Takeda, 1996 [40]	32.1 % (n = 28)	61.5 % (n = 13)	<0.05
Hu, 2009 [41]	38.1 % (n = 47)	10.4 % (n = 47)	<0.05
Namikawa, 2010 [42]	75.0 % (n = 10)	51.4 % (n = 24)	Not significant
Tokunaga, 2013 [43]	49.0 % (n = 79)	59.3 % (n = 68)	Not significant
Takeno, 2014 [44]	50.0 % (n = 14)	37.0 % (n = 58)	<0.05
Total	45.2 % (n = 180)	41.7 % (n = 210)	Not significant

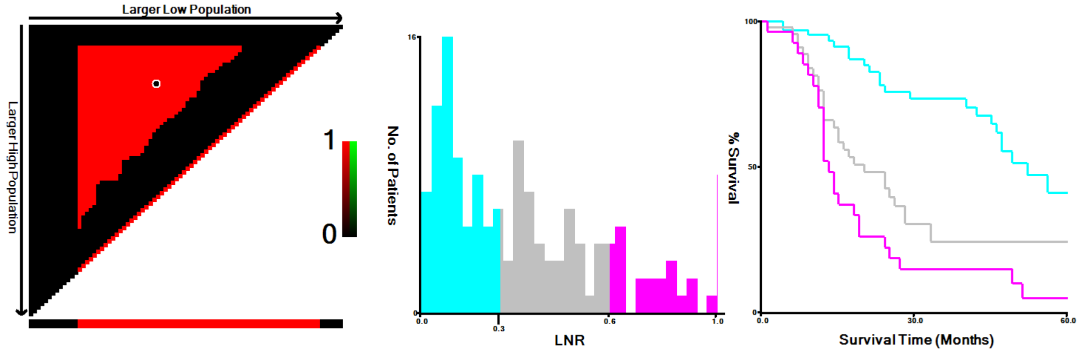

Supplemental Fig. 1 X-tile analysis identifying the best cutoff points for LNR; LNR, metastatic lymph node ratio.

A

AJCC7th staging	N0	N1	N2	N3
T1	IA	IB	IIA	IIB
T2	IB	IIA	IIB	IIIA
T3	IIA	IIB	IIIA	IIIB
T4a	IIB	IIIA	IIIB	IIIC
T4b	IIIB	IIIB	IIIC	IIIC

B

TRM staging	Nr0	Nr1	Nr2	Nr3
T1	IA	IB	IIA	IIB
T2	IB	IIA	IIB	IIIA
T3	IIA	IIB	IIIA	IIIB
T4a	IIB	IIIA	IIIB	IIIC
T4b	IIIB	IIIB	IIIC	IIIC

C

AJCC8th staging	N0	N1	N2	N3a	N3b
T1	IA	IB	IIA	IIB	IIIA
T2	IB	IIA	IIB	IIIA	IIIB
T3	IIA	IIB	IIIA	IIIB	IIIC
T4a	IIB	IIIA	IIIA	IIIB	IIIC
T4b	IIIA	IIIB	IIIB	IIIC	IIIC

Supplemental Fig. 2 (A) The AJCC 7th staging system, (B) the TRM staging system, (C) the AJCC 8th staging system

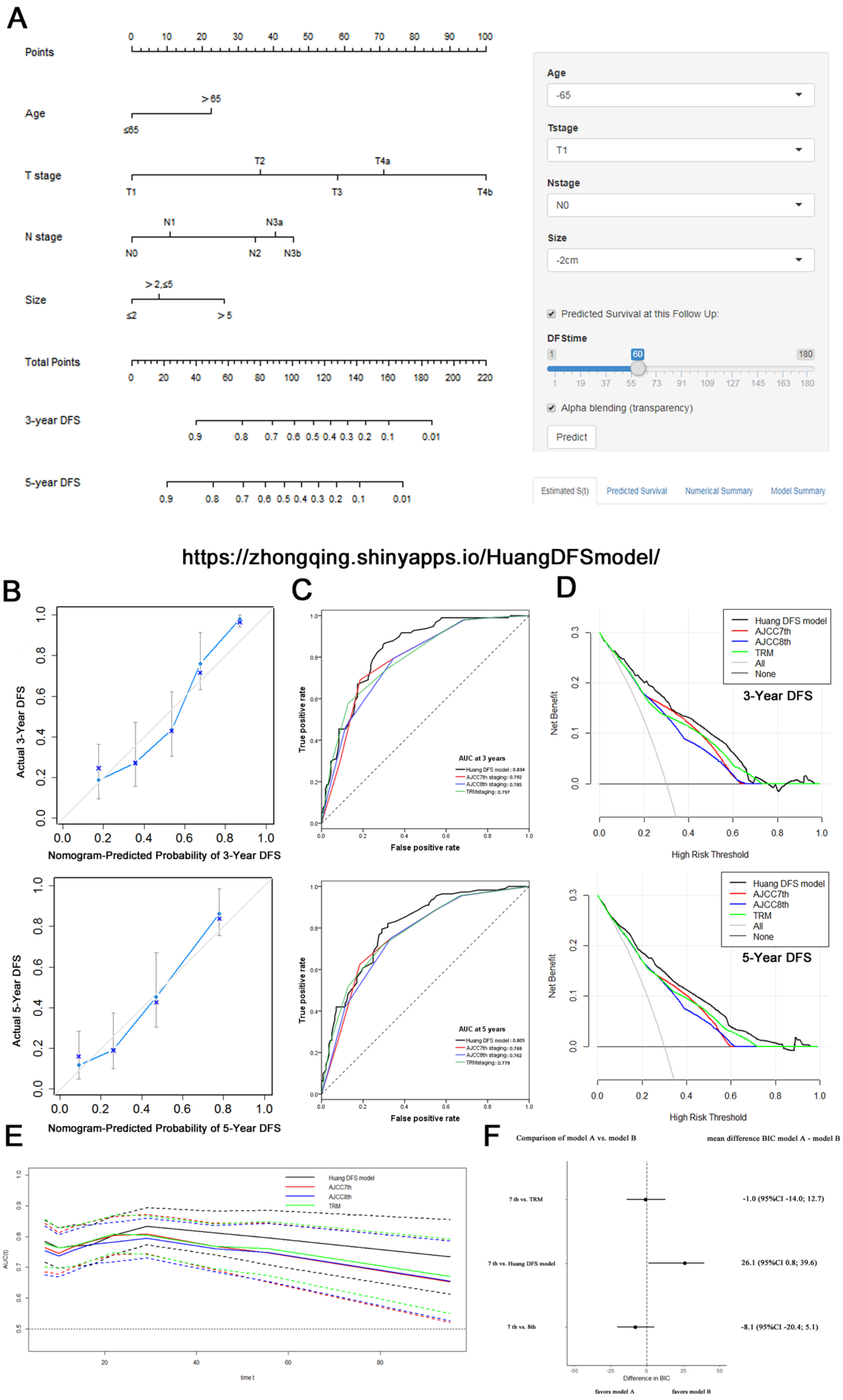
A
Points
Age
>65
≤65
T stage
T1
T2
T3
T4a
T4b
N stage
N0
N1
N2
N3a
N3b
Size
>2,≤5
≤2
>5
Total Points
3-year DFS
5-year DFS
Age
-65
Tstage
T1
Nstage
N0
Size
-2cm
Predicted Survival at this Follow Up:
DFStime
Alpha blending (transparency)
Predict
Estimated S(t)
Predicted Survival
Numerical Summary
Model Summary
https://zhongqing.shinyapps.io/HuangDFSmodel/
B
Actual 3-Year DFS
Nomogram-Predicted Probability of 3-Year DFS
Actual 5-Year DFS
Nomogram-Predicted Probability of 5-Year DFS
C
True positive rate
False positive rate
AUC at 3 years
AUC at 5 years
D
Huang DFS model
AJCC7th
AJCC8th
TRM
All
None
3-Year DFS
5-Year DFS
Net Benefit
High Risk Threshold
E
Huang DFS model
AJCC7th
AJCC8th
TRM
AUC(t)
time t
F
Comparison of model A vs. model B
mean difference BIC model A - model B
7 th vs. TRM
-1.0 (95%CI -14.0; 12.7)
7 th vs. Huang DFS model
26.1 (95%CI 0.8; 39.6)
7 th vs. 8th
-8.1 (95%CI -20.4; 5.1)
Difference in BIC
favors model A
favors model B

Supplemental Fig. 3 (A) The Huang DFS model (a web-based nomogram) for predicting the 3- and 5-year DFS rates for RGC. The nomogram is used by summing the points identified on the point scale for each variable. The total points projected on the bottom scales indicate the probability of 3- and 5-year DFS. The nomogram is available at https://zhongqing.shinyapps.io/Huang DFS model/. To use this nomogram, choose the value for each variable and the predicted survival time, then press the "predict" button. (B) A calibration plot of the web-based nomogram for 3 years and 5 years. (C) The receiver operating characteristic (ROC) curve for the 3- and 5-year disease-specific probability of the web-based nomogram and the 3 studied staging systems. (D) DCA for 3-year DFS and 5-year DFS after surgery. The y-axis measures the net benefit. (E) Time-dependent ROC curves for the web-based nomogram and the 3 studied staging systems. The x-axis represents the year after surgery, and the y-axis represents the estimated area under the ROC curve for survival at the time of interest. (F) The results from a bootstrap analysis (1,000 samples): mean differences in Bayesian information criteria (BIC) with 95% confidence limits for the web-based nomogram and the 3 studied staging systems.

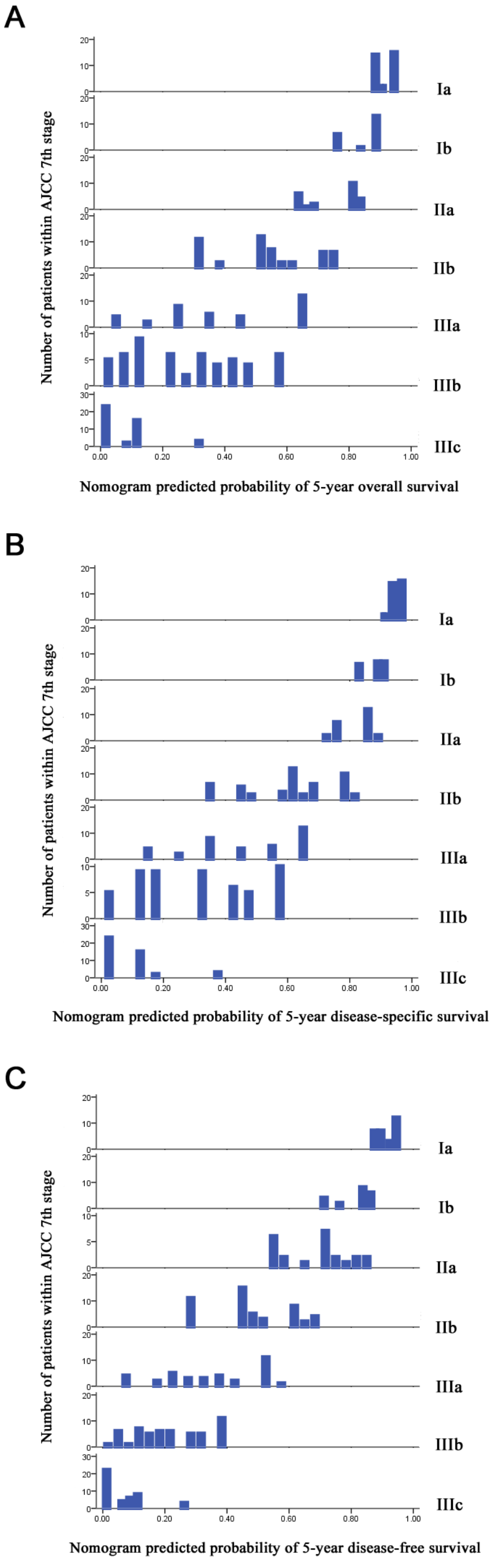

Supplemental Fig. 4 Distribution of the nomogram predictions within each AJCC 7th stage grouping. The nomogram's improved predictive ability is illustrated by the heterogeneity of the nomogram predictions. (A) overall survival, (B) disease-specific survival, (C) disease-free survival.

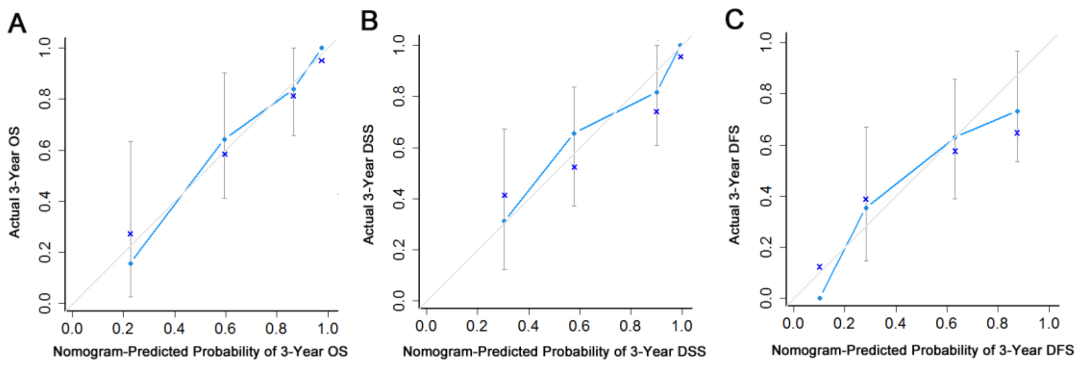

Supplemental Fig. 5 Nomogram calibration plots using the external validation cohort. (A) overall survival, (B) disease-specific survival, (C) disease-free survival.
